# Nutritional quality and greenhouse gas emissions of vegetarian and non-vegetarian primary school meals: A case study in Dijon, France

**DOI:** 10.3389/fnut.2022.997144

**Published:** 2022-10-10

**Authors:** Justine Dahmani, Sophie Nicklaus, Jean-Michel Grenier, Lucile Marty

**Affiliations:** ^1^Centre des Sciences du Goût et de l'Alimentation, CNRS, INRAE, Institut Agro, Université de Bourgogne Franche-Comté, Dijon, France; ^2^Direction de la restauration municipale et de l'alimentation durable de la Ville de Dijon, Dijon, France

**Keywords:** school canteen, meals, children, nutritional quality, greenhouse gas emissions, sustainability, vegetarian

## Abstract

Since 2018 in France, national regulation mandates that school canteens serve a weekly vegetarian meal to reduce school canteens' environmental impact in addition to previous regulations imposing nutritional composition guidelines. However, a lunch without meat is often perceived as inadequate to cover the nutritional needs of children. The present study aims to assess the nutritional quality and greenhouse gas emissions (GHGE) of vegetarian and non-vegetarian school meals served in primary schools in Dijon, France. The catering department provided the composition of 249 meals served in 2019. Nutritional content and GHGE were retrieved from national food databases. The portion size of each meal component was the standard portion size recommended by the relevant French authority (GEMRCN). Meals were classified into vegetarian meals, i.e., without meat or fish (*n* = 66), or non-vegetarian meals (*n* = 183). The nutritional adequacy of the meals for children aged from 6 to 11 years was estimated using the mean adequacy ratio (MAR/2,000 kcal) as the mean percentage of daily recommended intake for 23 nutrients and the mean excess ratio (MER/2,000 kcal) as the mean percentage of excess compared to the maximum daily recommended value for three nutrients. This analysis of actual school meals shows that both vegetarian and non-vegetarian meals had a similar good nutritional quality with MAR/2,000 kcal of 87.5% (SD 5.8) for vegetarian and of 88.5% (SD 4.5) for non-vegetarian meals, and a MER/2,000 kcal of 19.3% (SD 15.0) for vegetarian and of 19.1% (SD 18.6) for non-vegetarian meals. GHGE were more than twofold reduced in vegetarian compared to non-vegetarian meals (0.9 (SD 0.3) vs. 2.1 (SD 1.0) kgC0_2_ eq/meal). Thus, increasing the frequency of vegetarian meals, by serving egg-based, dairy-based or vegan recipes more frequently, would reduce GHGE while maintaining adequate nutritional quality of primary school meals.

## Introduction

Non-communicable diseases (e.g., overweight, obesity, diabetes, and cardiovascular diseases) as well as environmental threats (e.g., global warming, atmospheric pollution, water pollution and deforestation) require identifying dietary changes that will improve nutritional quality and reduce the environmental impact of diets (1, 2). Because school canteens may contribute to establishing social norms around eating and account for a significant share of food consumed by children, they could act as a lever toward more sustainable food systems, i.e. by making nutritious and environmentally friendly meals accessible to a large number of children through national or local public policies (3). Hence, modification of school catering taking environmental issues into account while maintaining a strong emphasis on nutrition now seems necessary (4–7) and possible, as optimization studies identified nutritionally adequate and environmentally friendly school meals (8, 9). In Spain, the municipality of Barcelona introduced low-carbon meals in public schools during the 2020–2021 school year. The evaluation of this experiment showed that the transition to a low-carbon meal had environmental benefits by halving the environmental impacts (10). In Bahia (Brazil), the Sustainable School Program (SSP) implemented low-carbon meals twice a week in 155 schools in 4 municipalities and showed a 17% reduction in diet-related greenhouse gas emissions (GHGE) (11).

Currently, in France, 8.5 million children aged 3 to 17 years eat at least once a week at school canteens. Among children aged 3 to 10 years, 58% eat lunch regularly at the school canteens, i.e., at least 4 days a week (12). In France, the responsibility for serving meals in primary schools lies with the municipalities. Meals can be provided by municipalities services or delegated to a catering company. Since the first “National Health and Nutrition Plan” (13) was launched in 2001 in France, primary school canteens have been targeted by public health measures. School meals are typically structured based on four or five components: starter (optional), protein dish, side dish, dairy product, and dessert (optional). Each day, a unique menu is proposed to children (14)^1^. In 2011, the Ministry of Agriculture published 15 mandatory recommendations based on work of the “Market Research Group for Collective Catering and Nutrition” (GEMRCN) related to the frequency of serving dishes over 20 consecutive meals and the portion sizes based on children's age (15)^2^. A study published in 2016 by Vieux et al. demonstrated that when the 15 French recommendations were met, a 20-meal sequence covered, on average, 36% of energy daily needs and 50% of essential nutrient needs for primary school children (16). A simulation based on a sample of 40 series of 20 meals showed that nutritional quality increased with the number of respected recommendations. When the recommendations were not followed, a risk of deterioration in the nutritional quality of meals emerged (16). This study highlighted that serving only vegetarian meals would decrease the nutritional quality of the meals served to children (16). However, this result may be partly due to the lack of variety in vegetarian dishes considered in this study (*n* = 41 among the 800 dish options).

In 2018, a law for the “balance of trade relations in the agricultural and food sector and sustainable healthy food accessible to all” (EGalim) was adopted in France (17)^3^. This law contains measures related to school catering that aim at promoting sustainable school meals by increasing the proportion of organic products up to 20%, limiting the use of plastic, preventing food waste, strengthening transparency and diversifying protein sources of meals with one vegetarian meal per week, i.e. without fish or meat (18)^4^. In 2021, the Climate and Resilience law amended the objectives of the EGalim law by encouraging more vegetarian meals up to a daily vegetarian option at primary school canteens (19)^5^. This context raises questions about the acceptability of vegetarian meals at school canteens in the French context, where meat has a central place in meal composition (20). The general council for food, agriculture and rural areas (governmental organization) issued an evaluation report on the weekly vegetarian school meal in March 2021 highlighting reluctance toward this measure that was perceived by part of the population as an attack on French tradition and gastronomy, in which vegetables usually appear as a side dish (21). The view that “a meal without meat is not a real meal” also seems to be widely shared among school catering actors. It also raises the question of potential degradation of nutritional quality, as suggested by Vieux et al. (16), although nutrient profiling methods have shown that vegetarian and non-vegetarian main dishes offered in primary schools in France were generally of good nutritional quality (22). In addition, a recent simulation study based on a database of 2,316 school dishes demonstrated that the best trade-off for decreasing the environmental impacts of school meals without altering their nutritional quality was a frequency of 12 vegetarian meals over a total of 20 meals (23).

Reduction of meat consumption in school canteens is key to building more environmentally friendly food systems, but evidence based on the analysis of school meals from real life is still needed to convince catering actors, parents and children of the nutritional quality of vegetarian meals. The challenge is thus to demonstrate that a high level of nutritional quality could be maintained while limiting the negative impacts of meals on the environment and, in particular, global warming.

In the present study, we used validated indicators to compare the nutritional quality and environmental impact of all meals (66 vegetarian and 183 non-vegetarian) that were served in 2019 to children aged from 6 to 11 years old in primary schools in Dijon, France. We hypothesized that vegetarian and non-vegetarian meals would have similarly good nutritional quality, whereas the environmental impact would be significantly reduced for vegetarian meals compared to non-vegetarian meals. Moreover, we aimed at analyzing more closely the nutritional quality and environmental impact of school meals based on the type of protein dish.

## Materials and methods

### Data collection and database information

This study focused on meals served in 2019 (January to December) to children in primary schools in Dijon, France (*n* = 249). In this context, school meals delivery is organized by the municipality service. The central kitchen of Dijon produces and serves 4,000 meals every day to children aged 6 to 11 years (plus 4,000 for preschool aged children and municipality staff) distributed among the 38 primary school canteens (24)^6^. Meals were developed by a dietician and respected the GEMRCN frequencies. The central kitchen provided the list of the food items included in each dish analyzed in this study. An example of a meal series for 1 month is available in the Supplementary Table S1.

Three databases were created from the collected information: the meal database, the dish database and the recipe database (data are available here: https://osf.io/fk7cq/). The meal database contained the 249 meals served in 2019 corresponding to five meals served per week (excluding weekend) during the school period and school holidays. A meal was composed of five or four components, among a starter, protein dish, side dish (e.g., fries, starches, vegetables or legumes), dairy product, and dessert, with a 30- or 40-g portion of bread. Meals were split into two main categories: vegetarian (*n* = 66, all components without meat or fish) and non-vegetarian (*n* = 183).

The dish database contained 434 distinct dishes, including 65 starters, 129 protein dishes, 60 sides, 69 dairy products and 71 desserts. Each dish had a name and a code. All dishes were classified as vegetarian (without meat or fish) or non-vegetarian. Moreover, the meals were categorized based on the subcategory of the protein dishes. Five different subcategories were defined based on their level of GHGE (25), from the most to the least emitting: beef, veal and lamb; pork and poultry; fish; eggs and/or cheese; and vegan (i.e., plant-based foods only). Each dish was composed of one of several food items (e.g., pork and curry sauce, eggs and mayonnaise).

The 433 food items were included in the recipe database and identified by a name and a code. The portion size of each food item (in grams) was established by the central kitchen and closely related to the GEMRCN recommendations for primary school children (15). All food items had an edible portion equal to one except for melon (0.7), banana (0.8), clementine (0.7), tangerine (0.7), orange (0.8) and chicken thigh (0.8).

The nutritional composition of each food item was estimated using two French food nutrient composition reference tables: CIQUAL 2020 and CALNUT 2020 (26)^7^. The GHGE (in kilograms of CO_2_ equivalent) of each food item was based on the AGRIBALYSE v3.0 table providing reference data on the environmental impacts of agricultural and food products obtained through life cycle analysis, comprising the following contributions to GHGE: agriculture, transport and packaging (27)^8^. Interoperability between the CIQUAL and AGRIBALYSE databases (same food products with same coding system) makes it possible to record both nutritional and environmental footprint information. A dietician performed the pairing of each food item from the central kitchen with items from these two databases. The confidence level of pairing was defined by rules available in the Supplementary Table S2. A total of 340 food items were paired at the highest confidence levels (one or two), representing approximately 79% of all recipes. For 224 food items, at least one nutrient was missing in the CIQUAL database, and imputed data from the CALNUT database were used.

### Nutritional quality and environmental footprint

Total weight, energy content, nutritional quality and environmental footprint indicators were calculated at the meal level. The environmental footprint was estimated by greenhouse gas emissions (GHGE, in kgCO_2_eq), the best-known and most-used climate change indicator (28, 29). This indicator corresponds to the modification of climate affecting the global ecosystem. The calculation was based on the theoretical portion sizes of the food items supplied by the central kitchen for children in primary school (6 to 11 years old). GHGE was calculated for each food item using Equation (1) and then summed at the dish level and at the meal level.


(1) $$\begin{array}{l} \begin{array}{lll} GHGE_{food\;item}\;(kgCO2eq) & = & portion\;(g)\times \end{array}\\ \;\begin{array}{l} \frac{GHGE_{Agribalyse}\;(kgC02eq/kg)}{1000\;(g)} \end{array} \end{array}$$

Similarly, the contents for 26 nutrients were calculated for each food item using Equation (2) and then summed at the dish level and at the meal level.


(2) $$\begin{array}{l} \begin{array}{lll} Nutrient\;content_{food\;item} & = & portion\;(g)\;\times \;edible\;portion\times \end{array}\\ \;\begin{array}{l} \frac{nutrient\;content\;CIQUAL\;(g/100g)}{100\;(g)} \end{array} \end{array}$$

To estimate the overall nutritional quality of the meal, we used the mean adequacy ratio (MAR) which estimates the average content of several nutrients expressed as a percentage of recommended intakes (16). In the present study, MAR was calculated by taking into account 23 nutrients (proteins, fibers, vitamins B1, B2, B3, B6, B9, B12, C, D, E, A, calcium, potassium, iron, magnesium, zinc, copper iodine, selenium, linoleic acid, alpha-linolenic acid, docosahexaenoic acid), expressed as the percentage of adequacy for 2,000 kcal of a meal, as indicated in Equation (3) (16). MAR/2,000 kcal represents the nutritional quality if a meal was scaled-up to provide the daily energy requirement of 2,000 kcal. The recommended daily intakes (RDI) for the 23 nutrients are available in the Supplementary Table S3 and were obtained by weighting the recommended dietary allowance in France for sex and age range (30) based on the age and sex representativeness in children aged 4–13 years attending primary school following the method proposed by Vieux et al. (16).


(3) $$\begin{array}{l} MAR/2000\;kcal\;=\;\frac{1}{23}\;\times \;\sum_{n=1}^{23}\frac{\frac{content_{n}}{nrj}\;\times 2000}{RDI_{n}}\;\times \;100 \end{array}$$

The MAR/2,000 kcal is reported on a scale from 0 to 100%, where 100% indicates that the daily requirements for all the nutrients were met. Each ratio was truncated to 1 so that a large quantity of one nutrient could not compensate for a small quantity of another, hence nutrient coverage beyond children's daily needs was not considered.

We also calculated the mean excess ratio (MER) which estimates the excess compared to the daily maximum recommended values (MRV) of three nutrients that should be limited: saturated fatty acids (SFA), salt and total sugars. MER was expressed as the percentage of excess compared to the MRV for 2,000 kcal of a meal, as indicated in Equation (4) (31). The MRV are available in the Supplementary Table S3 and were based on French recommendations for children aged 4–12 years (32).^9^, ^10^


(4) $$\begin{array}{l} \begin{array}{lll} MER/2000\;kcal & = & [\frac{1}{3}\times (\sum_{n=1}^{3}\frac{\frac{content_{n}}{nrj}\times 2000}{MRV_{n}}\times 100)] \end{array}\\ \;\begin{array}{ll} - & 100 \end{array} \end{array}$$

The MER/2,000 kcal is reported in percentage of excess, where 0% indicates that none of the three nutrient limits were reached. Each ratio was limited to 1, so that a small quantity of one nutrient could not compensate for a large quantity of another.

### Statistical analyses

Statistical analyses were conducted after computation of the data at the meal level, as explained above. Correlations between total weight of meals, energy content, GHGE, MAR/2,000 kcal and MER/2,000 kcal were computed with Pearson correlation coefficients. To compare vegetarian meals and non-vegetarian meals, two-sample Student's *t*-tests were performed on weight, energy, GHGE, MAR/2,000 kcal, MER/2,000 kcal and contents in 26 nutrients per 2,000 kcal of a meal. For each nutrient and each type of meal, we also compared the percentage of RDI or MRV for 2,000 kcal of meal to the target value (100%) using one-sample Student's *t*-tests. Then, we performed ANOVA models to compare outcome variables (weight, energy, GHGE, MAR/2,000 kcal, MER/2,000 kcal and the nutrient contents) between the five subcategories of meals based on their protein dish (beef, veal and lamb; pork and poultry; fish; eggs and/or cheese; and vegan) and pairwise *post hoc* comparisons were performed.

Statistical analyses were performed with SAS software version 9.4. The level of significance was set to 0.05 for all of the analyses and Bonferroni correction was used to control for multiple testing across the 26 nutrients (i.e., 0.05/26 = 0.002).

## Results

### Description of school meal nutritional quality and environmental impact

Data from 249 meals were considered for the present analysis. The average weight of a meal was 464 g (SD 64), and the average energy content was 659 kcal (SD 125) representing 33% of the recommended daily energy intake for children aged 6 to 11 years old (i.e., 2,000 kcal). The average greenhouse gas emissions (GHGE) of a meal was 1.8 kgCO_2_ eq (SD 1.0). The average MAR/2,000 kcal was 88.3% (SD 4.9), indicating that on average 88% of the RDI for the 23 nutrients would be covered by 2,000 kcal of a school meal. The average MER/2,000 kcal was 19.1% (SD 17.7) indicating that on average the MRV for three nutrients (SFA, salt and total sugars) would be exceeded by 19% with 2,000 kcal of a school meal.

Pearson's correlations showed a positive association between weight and energy content and a negative association between weight and MER/2,000 kcal highlighting that the content in nutrients to limit was lower in school meals of higher weight. There was also a negative association between energy content and MAR/2,000 kcal indicating that school meals of better nutritional quality were also those of lower energy content. No association was found between GHGE and weight, energy content nor nutritional quality of the meals (Table 1).

**Table 1:** Pearson's correlation coefficients between weight, energy content, GHGE, MAR/2,000 kcal and MER/2,000 kcal of the Dijon school meals in 2019 (*n* = 249).

		**GHGE**	**Energy content**	**MAR/2,000 kcal**	**MER/2,000 kcal**
Weight	*r* *p*	−0.06 0.307	0.27 < 0.001	0.01 0.86	−0.174 0.006
GHGE	*r* *p*		0.01 0.808	−0.001 0.993	0.004 0.955
Energy content	*r* *p*			−0.399 < 0.001	−0.053 0.411
MAR/2,000 kcal	*r* *p*				0.249 < 0.001

### Comparisons between non-vegetarian and vegetarian meals

Among the 249 meals served in 2019 in Dijon school canteens, 183 were non-vegetarian (73.5%), and 66 were vegetarian (26.5%), which is greater than the recommended frequency of one per week (i.e., 20%) established by the EGalim law. The nutritional quality and environmental impact of all meals (*n* = 249), non-vegetarian (*n* = 183) and vegetarian meals (*n* = 66) are shown in Table 2. Non-vegetarian and vegetarian meals had similar weights and energy contents. GHGE was significantly twofold reduced for vegetarian meals compared to non-vegetarian meals. On average, a vegetarian meal emitted 0.9 kgCO_2_eq (SD 0.3), whereas a non-vegetarian meal emitted 2.1 kgCO_2_eq (SD 1.0). MAR/2,000 kcal for non-vegetarian and vegetarian meals was not significantly different, likewise for MER/2,000 kcal.

**Table 2:** Nutritional quality and environmental impact of all meals (*n* = 249), non-vegetarian (*n* = 183) and vegetarian meals (*n* = 66) served in the Dijon school canteens in 2019.

	**RDI^1^ or MRV^2^**	**Mean (SD)**			**p^3^**
		**All meals** **(*n =* 249)**	**Non-vegetarian meals (*n =* 183)**	**Vegetarian meals (*n =* 66)**	
Weight (g)		464.0 (64)	463.2 (63.8)	466.3 (65.0)	0.734
Energy (%)	2,000 kcal	33.0 (6.2)	33.0 (6.3)	33.0 (5.9)	0.950
GHGE (kgCO2eq)		1.8 (1.0)	2.1 (1.0)	0.9 (0.3)	< 0.001
MAR/2,000 kcal (%)		88.3 (4.9)	88.5 (4.5)	87.5 (5.8)	0.152
MER/2,000 kcal (%)		19.1 (17.7)	19.1 (18.6)	19.3 (15.0)	0.921
Proteins (%RDI)	25 g	373.6 (107.3)	400.1 (105.2)	300.1 (74.2)	< 0.001
Fibers (%RDI )	13 g	215.0 (77.7)	199.1 (67.5)	259.1 (87.0)	< 0.001
Vitamin B1 (%RDI)	0.8 mg	132.5 (75.7)	137.3 (83.9)	118.9 (43.1)	0.090
Vitamin B2 (%RDI)	1.2 mg	105.8 (43.9)	106.8 (40.1)	102.9 (53.2)	0.544
Vitamin B3 (%RDI)	9 mg	180.7 (98.2)	206.2 (95.3)	109.7 (66.6)	< 0.001
Vitamin B6 (%RDI)	1 mg	174.3 (69.9)	182.1 (70.9)	152.4 (62.8)	0.002
Vitamin B9 (%RDI )	201 μg	181.6 (95.4)	160.4 (64.8)	240.4 (134.9)	< 0.001
Vitamin B12 (%RDI)	1.4 μg	325.2 (312.0)	387.8 (339.6)	151.5 (82.8)	< 0.001
Vitamin C (%RDI)	89 mg	**80.7 (63.9)**	85.2 (68.0)	**68.2 (49.3)**	0.065
Vitamin D (%RDI)	5 μg	87.5 (75.0)	92.3 (82.6)	**74.0 (45.5)**	0.089
Vitamin E (%RDI)	9.1 mg	141.1 (58.7)	133.0 (56.2)	163.6 (59.8)	< 0.001
Vitamin A^4^ (%RDI)	501 μg	286.5 (313.1)	291.5 (312.0)	272.4 (318.1)	0.676
Calcium (%RDI)	924 mg	**81.2 (27.1)**	**78.4 (26.6)**	**89.1 (27.3)**	0.007
Potassium (%RDI)	2892 mg	100.3 (25.3)	103.4 (25.9)	91.6 (21.5)	< 0.001
Iron (%RDI)	8.2 mg	122.0 (49.1)	119.5 (51.5)	129.1 (41.4)	0.174
Magnesium (%RDI)	203 mg	139.7 (42.1)	136.6 (42.1)	148.2 (41.1)	0.054
Zinc (%RDI)	9.2 mg	116.7 (58.5)	125.3 (64.9)	92.7 (21.9)	< 0.001
Copper (%RDI)	1.2 mg	181.8 (70.4)	176.7 (78.6)	196.0 (36.4)	0.056
Iodine (%RDI)	120 μg	206.5 (96.1)	213.5 (104.3)	187.2 (65.1)	0.057
Selenium (%RDI)	39 μg	577.0 (187.4)	583.3 (193.1)	559.5 (170.8)	0.352
LA^5^ (%RDI)	8.9 g	104.2 (44.3)	97.8 (44.0)	121.9 (40.3)	< 0.001
ALA^6^ (%RDI)	2.2 g	**59.3 (47.7)**	**62.8 (54.2)**	**49.8 (18.1)**	0.059
DHA^7^ (%RDI)	152 mg	138.6 (169.9)	156.7 (194.1)	88.6 (34.8)	0.005
SFA^8^ (%MRV)	26 g	**117.3 (48.6)**	**119.1 (49.9)**	**112.3 (44.6)**	0.306
Salt (%MRV)	6.5 g	**114.2 (38.8)**	**113.1 (40.4)**	**117.1 (34.0)**	0.445
Total sugars^9^ (%MRV)	67.5 g	84.5 (29.7)	81.2 (29.4)	93.5 (29.0)	0.004

^1^Recommended daily intake for children aged 4–13 years attending primary school in France (29). ^2^Maximum recommended value for children aged 4–12 years (31–33). ^3^Mean comparison of vegetarian and non-vegetarian meals (two-sample Student's *t*-test, significance: *p* < 0.05 for meal indicators, *p* < 0.002 for nutrients). ^4^Vitamin A = retinol + beta-carotene/6. ^5^Linoleic acid. ^6^Alpha-linolenic acid. ^7^Docosahexaenoic acid. ^8^Saturated fatty acids. ^9^Total sugars = fructose + glucose + maltose + saccharose. In bold, % RDI of nutrients lower than 100%, or % MRV of nutrients higher than 100% (one-sample Student's *t*-test, significance: *p* < 0.002), nutrients contents are given per 2,000 kcal of a meal.

Nineteen of 23 nutrients were found in non-limiting quantities (i.e., average content ≥100% RDI) in both vegetarian and non-vegetarian meals: proteins, fibers, vitamins B1, B2, B3, B6, B9, B12, E, A, potassium, iron, magnesium, zinc, copper, iodine, selenium, linoleic acid (LA) and docosahexaenoic acid (DHA) (Table 2). In contrast, the coverage of nutritional needs was insufficient (i.e., average content < 100%) for two nutrients in both types of meals: calcium and alpha-linolenic acid (ALA). Vitamin C and D were found in deficit specifically in vegetarian meals. Regarding nutrients to limit, the content was above the MRV and not statistically different in vegetarian and non-vegetarian meals for SFA (117.3% of MRV) and salt (114.2% of MRV). Values for total sugars was below the maximum limit for non-vegetarian (81.2%) and vegetarian meals (93.5%).

### Comparisons of the meals from the five protein dish subcategories

**Figure 1:**
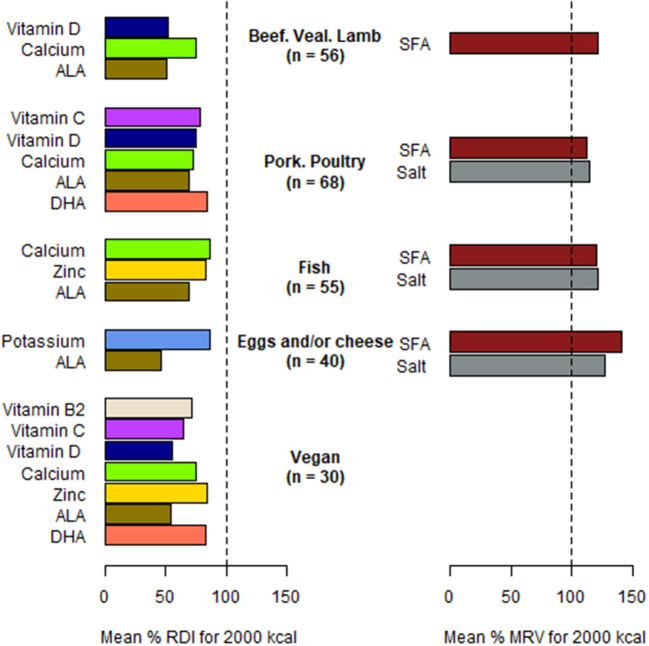
Nutrients in deficit and nutrients in excess in five meal subcategories based on protein dish served in Dijon primary school canteens in 2019. Nutrients in deficit are significantly below RDI, *p* < 0.002; Nutrients in excess are significantly above MRV, *p* < 0.002. RDI, recommended daily intake. MRV, maximum recommended value.

The nutritional quality and environmental impact across the five meal subcategories based on protein dish: beef, veal, lamb (*n* = 56); pork and poultry (*n* = 68); fish (*n* = 55); eggs and/or cheese (*n* = 40); and vegan (*n* = 30) are shown in Table 3. Meals had the same energy content across all subcategories [*F*_(4, 244)_ = 1.01, *p* = 0.404] despite different weights [*F*_(4, 244)_ = 4.40, *p* = 0.002]. Meals with vegan or fish-based main dishes had a significantly higher weight than meals with beef, veal or lamb dishes. A significant effect of the meal subcategory on GHGE [*F*_(4, 244)_ = 261.64, *p* < 0.001], MAR/2,000 kcal [*F*_(4, 244)_ = 3.81, *p* = 0.005] and MER/2,000 kcal [*F*_(4, 244)_ = 5.92, *p* < 0.001] was noted. GHGE differed between all five subcategories: meals with beef-, veal- or lamb-based dishes emitted the most GHGE followed by meals with fish-based dishes, meals with pork- or poultry-based dishes, meals with eggs and/or cheese-based dishes, and finally vegan dishes, with an approximately five-fold reduction compared to ruminant meat-based meals. MAR/2,000 kcal was significantly lower for meals with vegan dishes compared to meals with eggs and/or cheese or fish dishes, but the difference was small (4% difference between meals with vegan dishes compared to meals with eggs and/or cheese). MER/2,000 kcal was significantly lower for meals with vegan dishes compared to fish-based meals and meals with eggs and/or cheese dishes. Only meals with vegan dishes had SFA contents below the MRV [80.5% (SD 33.0)]. Each subcategory of meals had some nutrients below the RDI or above the MRV. Nutrients in deficit (significantly below RDI) and nutrients in excess (significantly above MRV) in the five meal subcategories are presented in Figure 1.

**Table 3:** Nutritional quality and environmental impact across the five meal subcategories based on protein dish: beef, veal, lamb (*n* = 56); pork and poultry (*n* = 68); fish (*n* = 55); eggs and/or cheese (*n* = 40); vegan (*n* = 30) served in the Dijon school canteens in 2019.

	**RDI^1^ or MRV^2^**	**Mean (SD)**					**p^3^**
		**Beef. Veal. lamb** **(*n =* 56)**	**Pork. poultry** **(*n =* 68)**	**Fish** **(*n =* 55)**	**Eggs and/or cheese (*n =* 40)**	**Vegan** **(*n =* 30)**	
Weight (g)		439.5 (66.4)^b^	472.2 (63.9)^ab^	479.5 (54.2)^a^	450.1 (60.4)^ab^	481.5 (67.0)^a^	0.002
Energy (%)		32.4 (6.3)^a^	34.2 (6.5)^a^	32.4 (6.1)^a^	32.6 (6.9)^a^	32.6 (4.4)^a^	0.404
GHGE (kgCO2eq)		3.4 (0.8)^a^	1.3 (0.4)^c^	1.8 (0.4)^b^	1.0 (0.3)^d^	0.7 (0.2)^e^	< 0.001
MAR/2,000 kcal (%)		87.6 (3.2)^ab^	88.4 (4.4)^ab^	89.2 (5.3)^a^	89.6 (6.8)^a^	85.6 (3.8)^b^	0.005
MER/2,000 kcal (%)		15.6 (14.8)^bc^	19.2 (19.8)^abc^	20.9 (19.2)^ab^	28.4 (15.0)^a^	9.9 (11.1)^c^	< 0.001
Proteins (%RDI)	25 g	386.9 (85.2)^a^	408.7 (110.6)^a^	405.8 (118.7)^a^	335.8 (75.6)^ab^	260.7 (47.3)^b^	< 0.001
Fibers (%RDI )	13 g	204.8 (66.3)^b^	197.0 (73.3)^b^	196.2 (64.2)^b^	230.7 (91.5)^b^	288.5 (66.2)^a^	< 0.001
Vitamin B1 (%RDI)	0.8 mg	121.6 (65.4)^a^	164.8 (111.9)^a^	118.8 (46.5)^a^	120.8 (50.2)^a^	120.1 (33.2)^a^	0.001
Vitamin B2 (%RDI)	1.2 mg	111.5 (40.3)^ab^	104.8 (40.3)^ab^	98.6 (33.6)^bc^	135.0 (55.5)^a^	**71.5 (29.4)** ^c^	< 0.001
Vitamin B3 (%RDI)	9 mg	188.6 (46.7)^b^	270.5 (106.9)^a^	149.8 (71.1)^b^	132.3 (74.4)^bc^	83.1 (34.0)^c^	< 0.001
Vitamin B6 (%RDI)	1 mg	195.5 (58.5)^a^	197.1 (78.6)^a^	151.2 (65.3)^a^	152.4 (49.4)^a^	154.0 (75.2)^a^	< 0.001
Vitamin B9 (%RDI )	201 μg	154.5 (53.4)^bc^	152.3 (71.2)^c^	168.8 (61.4)^bc^	256.3 (151.3)^a^	222.9 (98.8)^ab^	< 0.001
Vitamin B12 (%RDI)	1.4 μg	418.7 (182.9)^b^	160.9 (56.1)^c^	635.5 (470.3)^a^	215.3 (72.9)^c^	100.7 (105.7)^c^	< 0.001
Vitamin C (%RDI)	89 mg	90.1 (69.1)^a^	**78.6 (59.0)** ^a^	82.6 (74.1)^a^	80.8 (56.9)^a^	**64.1 (52.4)** ^a^	0.502
Vitamin D (%RDI)	5 μg	**51.4 (35.0)** ^b^	**74.9 (45.0)** ^b^	152.4 (113.9)^a^	94.4 (50.9)^b^	**55.0 (36.5)** ^b^	< 0.001
Vitamin E (%RDI)	9.1 mg	125.8 (52.8)^a^	125.5 (54.0)^a^	148.9 (58.4)^a^	169.7 (65.3)^a^	152.6 (54.1)^a^	< 0.001
Vitamin A^4^ (%RDI)	501 μg	291.1 (316.0)^a^	289.8 (321.8)^a^	283.5 (291.6)^a^	339.5 (391.6)^a^	204.9 (185.4)^a^	0.527
Calcium (%RDI)	924 mg	**74.9 (26.4)** ^b^	**72.6 (22.3)** ^b^	**86.1 (26.4)** ^ab^	102.7 (28.6)^a^	**75.0 (21.6)** ^b^	< 0.001
Potassium (%RDI)	2892 mg	102.6 (23.8)^a^	103.6 (24.9)^a^	104.7 (29.8)^a^	**85.8 (20.5)** ^a^	99.3 (19.8)^a^	0.002
Iron (%RDI)	8.2 mg	147.7 (66.4)^a^	105.7 (31.2)^b^	102.2 (37.3)^b^	134.0 (48.5)^ab^	131.2 (36.4)^ab^	< 0.001
Magnesium (%RDI)	203 mg	122.8 (30.4)^b^	134.7 (38.1)^ab^	152.2 (50.8)^ab^	140.5 (46.3)^ab^	158.3 (32.8)^a^	< 0.001
Zinc (%RDI)	9.2 mg	188.5 (75.1)^a^	108.8 (35.2)^b^	**82.3 (22.6)** ^b^	100.8 (24.7)^b^	**84.5 (14.8)** ^b^	< 0.001
Copper (%RDI)	1.2 mg	170.8 (35.7)^a^	167.0 (35.6)^a^	192.7 (132.0)^a^	182.5 (34.7)^a^	215.0 (28.5)^a^	0.014
Iodine (%RDI)	120 μg	159.8 (56.9)^b^	171.8 (50.2)^b^	315.3 (119.2)^a^	186.5 (76.0)^b^	199.7 (51.7)^b^	< 0.001
Selenium (%RDI)	39 μg	495.1 (165.6)^c^	543.9 (157.1)^bc^	724.7 (187.7)^a^	475.1 (130.6)^c^	669.9 (150.3)^ab^	< 0.001
LA^5^ (%RDI)	8.9 g	95.6 (51.7)^b^	109.8 (37.5)^ab^	84.9 (38.8)^b^	112.9 (31.6)^ab^	131.6 (49.8)^a^	< 0.001
ALA^6^ (%RDI)	2.2 g	**50.9 (40.8)** ^a^	**69.1 (59.7)** ^a^	**68.6 (59.2)** ^a^	**45.7 (19.3)** ^a^	**54.1 (15.0)** ^a^	0.034
DHA^7^ (%RDI)	152 mg	87.0 (177.7)^b^	**83.8 (32.9)** ^b^	318.4 (234.3)^a^	98.5 (46.2)^b^	**83.3 (23.8)** ^b^	< 0.001
SFA^8^ (%MRV)	26 g	**121.5 (47.5)** ^**a**^	**113.1 (47.3)** ^**ab**^	**120.8 (53.9)** ^**a**^	**141.7 (37.5)** ^**a**^	80.5 (33.0)^b^	< 0.001
Salt (%MRV)	6.5 g	100.9 (32.5)^a^	**114.7 (48.0)** ^**a**^	**121.7 (34.3)** ^**a**^	**127.1 (30.1)** ^**a**^	106.7 (37.5)^a^	0.006
Total sugars^9^ (%MRV)	67.5 g	83.0 (29.0)^a^	85.7 (30.4)^a^	74.9 (27.7)^a^	92.1 (33.9)^a^	91.9 (23.2)^a^	0.032

^1^Recommended daily intake for children aged 4–13 years attending primary school in France (29). ^2^Maximum recommended value for children aged 4–12 years (31–33). ^3^Type III fixed effects tests of the protein dish subcategory effect in ANOVA models with weight, GHGE, MAR/2,000 kcal, MER/2,000 kcal, energy content and nutrient content as dependent variables. The same numbers indicate no significant difference between subcategories (*post hoc* pairwise comparisons, significance: *p* < 0.05 for meal indicators, *p* < 0.002 for nutrients). ^4^Vitamin A = retinol + beta-carotene/6. ^5^Linoleic acid. ^6^Alpha-linolenic acid. ^7^Docosahexaenoic acid. ^8^Saturated fatty acids. ^9^Total sugars = fructose + glucose + maltose + saccharose. In bold, % RDI of nutrients lower than 100%, or % MRV higher than 100% (one-sample Student's *t*-test„ significance: *p* < 0.002), nutrients contents are given per 2,000 kcal of a meal.

Comparative analyses between non-vegetarian and vegetarian meals as well as the five meal subcategories based on protein dish were replicated using MAR, MER and nutritional values per meal (instead of per 2,000 kcal) to present the net intake per meal. These tables are available in Supplementary Tables S4,S5.

## Discussion

The goal of the present study was to compare the nutritional quality and environmental impact of vegetarian and non-vegetarian meals served in Dijon primary school canteens in 2019. Based on the national regulation that encourages a weekly vegetarian meal in school canteens, the school catering department of Dijon exceeded the recommendation in 2019 with greater than one-quarter of all meals being vegetarian meals. The average greenhouse gas emissions of a meal (1.8 kgCO_2_eq) were consistent with results found in previous studies: 1.7 kgCO_2_eq (33), 1.0 kgCO_2_eq (34), and 1.4 kgCO_2_eq (35). The GHGE of non-vegetarian meals was on average greater than two-fold higher than the GHGE of vegetarian meals. Meals with beef, veal or lamb dishes had the highest levels of GHGE followed by meals with fish dishes, meals with pork or poultry dishes, meals with eggs and/or cheese and finally vegan meals. MAR/2,000 kcal and MER/2,000 kcal of non-vegetarian and vegetarian meals were not significantly different, indicating similar adequacy with recommended daily intakes in 23 nutrients to favor and with maximum recommended values in three nutrients to limit. However, MAR/2,000 kcal and MER/2,000 kcal were significantly lower for meals with vegan dishes compared to meals with fish dishes or eggs and/or cheese dishes indicating that meals with vegan dishes had more deficits in beneficial nutrients but also less excess in harmful nutrients. For all subcategories of meals, the protein content was high, as 2,000_kcal of a meal could cover between 260.7 and 408.7% of the RDI. This finding highlights rooms for reduction of protein dish size, which would also lower GHGE. Based on the French RDI, we highlighted that nutrient deficits are generally specific to each subcategory of dishes, except for ALA content which was below the RDI for all categories of meals and calcium content which was also below the RDI for all categories of meals except eggs and/or cheese subcategory.

On average, a meal provided 659 (SD 124) kcal. This result is similar to values found in previous studies investigating the nutritional quality of school meals in France: 712 kcal (16) and 590 kcal (36). Our results confirmed the nutritional adequacy of the primary school meals served during a year in one French city; such adequacy was previously reported based on meal composition simulations (16, 22). We thus demonstrated that the willingness of French policy-makers to provide nutritionally adequate meals in school canteens (14, 18) translated into concrete results in the school.

Our data provide specific insight into how the nutritional needs of children are covered in a given school canteen organization. Based on the consumption of 7- to 10-year-old children from the French INCA3 study, some nutrients (fibers, LA, ALA, DHA, iron, vitamin D and vitamin E) have been identified as not reaching the recommendations defined by EFSA (12). Based on our results, ALA RDI were also insufficiently covered in most meals and thus dietitians may be specifically encouraged to include ALA-rich foods when developing school menus. This could be achieved without relying on supplementation, for example by including more seeds and oils with high level of omega-3. A focus on salt and SFA contents reduction would be needed as they exceeded the recommended limit in most meals. Only meals with vegan dishes did not excess the recommendation for salt and SFA and in this respect they may be served more frequently. However, this may call for a reformulation of vegan dishes to increase their beneficial nutrient densities. Overall, differences in nutrients content across the five meal subcategories were small and they provided a variety of specific nutrients, leading to high MAR/2,000 kcal values (from 85.6 to 89.6%) but for different reasons. Moreover, the five meal subcategories provided different nutrients for which coverage was higher than the RDI which could compensate for deficit in the same nutrient in other type of meals, e.g., fish-based meals were particularly rich in vitamin D [152.4% RDI (SD 113.9)] and DHA [318.4% RDI SD (234.3)]. Fish-based meals could then be encouraged to promote vitamin D and DHA intake, while ensuring fish products come from sustainable fisheries. Aquaculture generally generates less GHGE than animal farming (37) but it requires substantial energy resources and generates water pollution (38). These findings highlight the complementarity of meal subcategories in reaching overall nutritional adequacy.

Some nutrients were found in low quantities in school meals compared to the nutritional intake recommendations for children, but others excessively covered dietary needs. In particular, the protein content was high for vegetarian meals (300.1% of daily recommended intake for 2,000 kcal), even for meals with vegan-dishes based meals (260.7%); not to mention the skyrocketing rate in non-vegetarian meals (400.1%). Thus, based on our data, most school lunches cover daily protein needs in excess (>100%).

No associations were found between GHGE and the weight or energy content of the meals. This result differs from those of a study examining the relationship between the environmental impact and nutrient content of sandwiches and drinks sold in a university canteen (UK), showing that the environmental impact score was positively associated with portion sizes and calories (39). This difference may be explained by the fact that the school meal system established in France (e.g., four or five components per meal with guidelines regarding frequencies of food groups and portion sizes) strongly determines weight and energy content, thus limiting their variability but not that of GHGE.

Nor the correlation between GHGE and MAR/2,000 kcal, neither between GHGE and MER/2,000 kcal were significant. We showed that non-vegetarian meals emitted more GHGE than vegetarian meals and that the nutritional quality of non-vegetarian and vegetarian meals was similar, which may partly explain the lack of association between GHGE and indicators of nutritional quality. In a context where reducing GHGE from the food system is necessary and urgent to meet the Paris Agreement's goal of limiting the increase in global temperature to 1.5 or 2°C (40), we highlighted that lowering GHGE of school meals can be done without damaging consequences on children's health. Consistent with this idea, the results of a recent study showed that the best trade-off would be a series of 20 meals with 12 vegetarian, four fish-based and four pork- or poultry-based meals (23). Beyond school meals, recent evidence suggested that there are no specific nutritional risk in vegetarian children (41) and no clinically meaningful differences in growth or biochemical measures in vegetarian children (42).

### Strengths and limitations

The present study has several limitations. First, we used nutritional and environmental indicators from CIQUAL, CALNUT and AGRIBALYSE databases that include values on average food items. We evaluated meal environmental impact based on GHGE but we did not use other indicators such as eutrophication, acidification, toxicity, biodiversity which may encompass other important impacts on our planet. Moreover, one must note that AGRIBALYSE database did not provide a distinction between organic and conventional food items, although their impacts may differ. For nutritional evaluation, we did not consider actual food items composition but average food items composition from the French reference databases. Nutrient content may vary especially for salt and fat depending on culinary practices. As the dietician from the central kitchen reported trying to limit the salt and fat content of the meals, the MER may have been overestimated for some recipes. The confidence levels for pairing with average food items were mostly high. For 92 items out of 433 with the lowest confidence levels, it would have been interesting to retrieve actual food items composition to estimate nutrient content more precisely.

The bioavailability of nutrients was not considered in the nutritional evaluation. The bioavailability of iron and zinc has been showed to be lower when they come from plant-based products (43). Bioavailability of iron (especially non-haem iron) and zinc is altered by the presence of phytates present in certain fiber-rich plants such as whole grains and pulses (44). However, bioavailability of zinc is moderately impacted even for low animal-to-plant ratio, whereas iron absorption is affected by an increase of plant products in the diet (45).

Another limitation is that we considered the food portions used by the central kitchen to estimate production needs (closely related to the GEMRCN recommendations) to calculate the nutritional quality indicators. However, the portions established by the central kitchen were probably different from the portions actually served to children and different from the portions actually consumed by children. Therefore, it would be needed to evaluate the portions of each component consumed by the children to estimate the variability in coverage of individual nutritional needs. This could be achieved by using individual food intake measure methods used in previous studies in children (46, 47). This would also provide useful insights to revise the size of the portions served as a protein dish, which may help limit food waste.

This study has several strengths. First, it was based on a unique database of meals served in one primary school canteen system during a one-year period. For the first time, the nutritional quality and GHGE were conjointly estimated for actual meals within one system. Based on the availability of yearly data, further studies could examine the evolution of nutritional quality and GHGE over several periods or seasons throughout the year or over several years. More globally, in the context of a current initiative of the Dijon catering system toward more sustainable food systems, these data provide a baseline estimation to follow the evolution of Dijon school catering during the coming years. Moreover, integrating other indicators reflecting other dimensions of sustainable food systems (48) such as attendance at school canteens, food waste, meal cost and children's liking of meals, could provide a holistic view to go toward more sustainable school meals.

## Conclusion

In this study, we assessed the nutritional quality and GHGE of meals served in 2019 in Dijon primary school canteens. We showed that all meals were of good nutritional quality, notably as 2,000 kcal of a meal could cover on average 88% of the recommended daily needs for 23 nutrients. Vegetarian meals (i.e., without meat or fish) had on average two-fold lower GHGE compared with non-vegetarian meals. Thus, increasing the frequency of vegetarian meals beyond the current regulation (one per week) seems to be a good strategy to meet the double challenge of maintaining good nutritional quality and reducing the carbon impact of school catering. The school catering system of Dijon, like other municipalities, could integrate more vegetarian meals by increasing the frequency of dishes based on eggs, dairy products or vegan recipes. Future research work aimed at improving the sustainability of school catering, which would be nutritionally adequate for children and respectful of the environment, should ensure that children's habits and tastes, costs for families and for farmers are also taken into account.

## Data availability statement

The datasets presented in this study can be found in online repositories. The names of the repository/repositories and accession number(s) can be found below: https://osf.io/fk7cq/.

## Author contributions

Conceptualization, methodology, and writing—review and editing: JD, LM, and SN. Formal analysis and writing—original draft preparation: JD. Resources: J-MG. Data curation: JD and LM. Supervision: LM and SN. Funding acquisition: SN. All authors contributed to the article and approved the submitted version.

## Funding

This research was funded by a grant to SN (Danone International Prize on Alimentation 2018, grant #DA20180125001), a grant from the Dijon Municipality in the frame of the Dijon Alimentation Durable 2030 initiative (grant #00006226-29dmact11), which supported the PhD fellowship of JD, and a grant of the Caisse des dépôts et consignations through the Territoires d'innovation funding scheme (grant #00005065). Funders were not involved in the data analysis plan.

## Conflict of interest

The authors declare that the research was conducted in the absence of any commercial or financial relationships that could be construed as a potential conflict of interest.

## Publisher's note

All claims expressed in this article are solely those of the authors and do not necessarily represent those of their affiliated organizations, or those of the publisher, the editors and the reviewers. Any product that may be evaluated in this article, or claim that may be made by its manufacturer, is not guaranteed or endorsed by the publisher.

## Footnotes

^1^ https://www.education.gouv.fr/la-restauration-scolaire-6254

^2^ https://www.economie.gouv.fr/daj/recommandation-nutrition

^3^ https://www.legifrance.gouv.fr/loda/id/JORFTEXT000037547946/

^4^ https://agriculture.gouv.fr/egalim-tout-savoir-sur-la-loi-agriculture-et-alimentation

^5^ https://ma-cantine.beta.gouv.fr/blog/10/

^6^ http://www.dijon.fr/Dijon-au-quotidien/Enfance-petite-enfance/Cantine-Inscription

^7^ https://ciqual.anses.fr/

^8^ https://doc.agribalyse.fr/documentation-en/

^9^ANSES. *Le sel* (2012). Available online at: https://www.anses.fr/fr/content/le-sel (accessed September 1, 2022).

^10^ANSES. *Les lipides* (2021). Available online at: https://www.anses.fr/fr/content/les-lipides (accessed September 1, 2022).

## Abbreviations

ALA: alpha-linolenic acid
DHA: docosahexaenoic acid
GHGE: greenhouse gas emissions
MAR: mean adequacy ratio
MER: mean excess ratio
MRV: maximum recommended value
LA: linoleic acid
RDI: recommended daily intake
SFA: saturated fatty acids
SSP: sustainable school program.
