# Supplementary material for: Nutritional quality and greenhouse gas emissions of vegetarian and non-vegetarian primary school meals: A case study in Dijon, France
Source: Front Nutr. 2022 Oct 10;9:997144. doi: 10.3389/fnut.2022.997144 (PMC9590375; doi:10.3389/fnut.2022.997144)
Supplement: Supplementary file 1 [file Table_1.DOCX]

Supplementary Material

# Supplementary Tables

**Table S1**. Examples of meals served in Dijon school canteens in March 2019 (FR)

| **Monday 4** | **Tuesday 5** | **Wednesday 6** | **Thursday 7** | **Friday 8** |
| --- | --- | --- | --- | --- |
| Gougères | Celeriac | Asparagus / mayonnaise | Lettuce | Tomatoes vinaigrette |
| Grilled ham | Fish | Soyballs with tomato sauce | Beef with gingerbread sauce | Chicken |
| Green beans | Mashed potatoes | Rice | Pasta | Cauliflower |
| Cheese | Vanilla custard | Cheese | Strawberry yogurt drink | Cheese |
| Apple | - | Banana | - | Donut |
|  |  |  |  |  |
| **Monday 11** | **Tuesday 12** | **Wednesday 13** | **Thursday 14** | **Friday 15** |
| Lettuce | Lettuce | Cheese pie | Shredded carrots | Fresh vegetable soup |
| Fish | Omelette | Lamb curry | Cheese croquette | Roasted pork with juice |
| Spinach | Ratatouille | Brocoli flan | Lentils | Fries |
| Cottage cheese | Cheese | Cheese | Fruit yogurt | Cheese |
| Orange | Basque cake | Peer | - | Pineapple |
|  |  |  |  |  |
| **Monday 18** | **Tuesday 19** | **Wednesday 20** | **Thursday 21** | **Friday 22** |
| Lettuce | Beetroot | Tomatoes vinaigrette | Radish salad | Tabbouleh |
| Cheese pastry with cream sauce | Roasted chicken | Veal | Sauerkraut | Fish |
|  | Quinoa | Carrots | Sausage and ham | Mixed vegetables |
| Cottage cheese | Cheese | Cheese | Yogurt | Cheese |
| Apple purée | Clementine | Praline cake | Madeleine | Clementine |
|  |  |  |  |  |
| **Monday 25** | **Tuesday 26** | **Wednesday 27** | **Thursday 28** | **Friday 29** |
| Lettuce | Cucumber | Lettuce | Cabbage and carrots | Beetroot |
| Spaghetti and bolognese sauce | Fish | Chickpeaballs with tomato sauce | Beef | Poached eggs |
|  | Mixed vegetables | Indian style cereals | Zucchini | Wheat |
| Vanilla yogurt | Cheese | Chocolate custard | Cottage cheese | Cheese |
| - | Flan | - | Waffle with strawberry jam | Fruit salad |

**Table S2**. Confidence levels of pairing with CIQUAL/AGRIBALYSE food items

| Confidence level | Rules | n |
| --- | --- | --- |
| 1 | Exact same label  Same food item but different label (e.g., brand name) | 133 |
| 2 | Similar label but differences on: preservation method (e.g., fresh/frozen), cooking method or packaging  Similar label but ingredient or fragrance variation (e.g., strawberry/vanilla ice cream)  More generic label (e.g., type of food item: blue cheese) | 207 |
| 3 | Similar label but ingredient missing or additional ingredient  Similar label but raw vs. cooked difference  More generic label (e.g., small food group: leaf vegetables)  More than one level-2 rules | 75 |
| 4 | More generic label (large food group: vegetables) | 17 |

**Table S3**. Nutrients used for MAR/2000 kcal (n=23) and MER/2000 kcal (n=3) calculation and corresponding recommended daily intakes (RDI) or maximum recommended values (MRV).

| **Nutrient** | **Unit** | **RDI**^1^ **or** **MRV**^2^ |
| --- | --- | --- |
| Energy | kcal | 2000 |
| Proteins | g | 25 |
| Fibers | g | 13 |
| Vitamin B1 | mg | 0.8 |
| Vitamin B2 | mg | 1.2 |
| Vitamin B3 | mg | 9 |
| Vitamin B6 | mg | 1 |
| Vitamin B9 | μg | 201 |
| Vitamin B12 | μg | 1.4 |
| Vit C | mg | 89 |
| Vit D | μg | 5 |
| Vit E | mg | 9.1 |
| Vit A^3^ | μg | 501 |
| Iode | μg | 120 |
| Calcium | mg | 924 |
| Potassium | mg | 2892 |
| Iron | mg | 8.2 |
| Magnesium | mg | 203 |
| Zinc | mg | 9.2 |
| Copper | mg | 1.2 |
| Iodine | μg | 120 |
| Selenium | μg | 39 |
| LA^4^ | g | 8.9 |
| ALA^5^ | g | 2.2 |
| DHA^6^ | mg | 152 |
| SFA^7^ | g | 26 |
| Salt | g | 6.5 |
| Total sugars^8^ | g | 67.5 |

^1^ Recommended daily intake for children aged 4–13 years attending primary school in France (Martin, 2001; Vieux *et al.*, 2016; EnScol *et al.*, 2020). ^2^ Maximum recommended value for children aged 4-12 years (ANSES, 2012, 2019a, 2021b). ^3^Vitamin A = retinol + beta-carotene/6. ^4^Linoleic acid. ^5^Alpha-linolenic acid. ^6^ Docosahexaenoic acid. ^7^Saturated fatty acids. ^8^Total sugars = fructose + glucose + maltose + saccharose

**Table S4**. MAR and nutrient content per meal for all meals (n=249), non-vegetarian (n = 183) and vegetarian (n = 66) meals served in the Dijon school canteens in 2019

|  |  | **Mean (SD)** | | |  |
| --- | --- | --- | --- | --- | --- |
|  | **RDI**^1^ **or MRV**^2^ | **All meals  (n=249)** | **Non-vegetarian meals**  **(n=183)** | **Vegetarian meals (n=66)** | ***p^3^*** |
| MAR (%) |  | 48.7 (6.4) | 49.4 (6.4) | 46.9 (5.8) | 0.003 |
| Proteins (g/meal) | 25 | 30.1 (7.8) | 32.2 (7.3) | 24.5 (6.3) | <0.001 |
| Fibres (g/meal) | 13 | 9.0 (3.2) | 8.3 (2.7) | 10.9 (3.7) | <0.001 |
| Vitamin B1 (mg/meal) | 0.8 | 0.3 (0.2) | 0.4 (0.2) | 0.3 (0.1) | 0.016 |
| Vitamin B2 (mg/meal) | 1.2 | 0.4 (0.1) | 0.4 (0.1) | 0.4 (0.2) | 0.409 |
| Vitamin B3 (mg/meal) | 9 | 5.3 (3.0) | 6.1 (2.9) | 3.2 (2.0) | <0.001 |
| Vitamin B6 (mg/meal) | 1 | 0.6 (0.2) | 0.6 (0.2) | 0.5 (0.2) | 0.001 |
| Vitamin B9 (μg/meal) | 201 | 117.5 (30.6) | 104.0 (42.1) | 155.0 (84.2) | <0.001 |
| Vitamin B12 (μg/meal) | 1.4 | 1.5 (1.5) | 1.8 (1.6) | 0.7 (0.4) | <0.001 |
| Vitamin C (mg/meal) | 89 | 22.5 (16.9) | 23.7 (17.9) | 19.3 (13.4) | 0.036 |
| Vitamin D (μg/meal) | 5 | 1.4 (1.3) | 1.5 (1.4) | 1.2 (0.7) | 0.015 |
| Vitamin E (mg/meal) | 9.1 | 4.2 (1.9) | 4.0 (1.9) | 4.9 (1.9) | 0.001 |
| Vitamin A^4^ (μg/meal) | 501 | 452.1 (473.4) | 459.0 (461.0) | 433.1 (509.6) | 0.717 |
| Calcium (mg/meal) | 924 | 243.5 (82.2) | 235.0 (80.3) | 267.2 (83.4) | 0.006 |
| Potassium (mg/meal) | 2892 | 938.7 (248.9) | 970.4 (262.0) | 851.1 (182.7) | <0.001 |
| Iron (mg/meal) | 8.2 | 3.2 (1.2) | 3.2 (1.3) | 3.4 (0.9) | 0.107 |
| Magnesium (mg/meal) | 203 | 91.1 (24.7) | 88.8 (23.0) | 97.5 (28.3) | 0.028 |
| Zinc (mg/meal) | 9.2 | 3.5 (1.7) | 3.7 (1.9) | 2.8 (0.6) | <0.001 |
| Copper (mg/meal) | 1.2 | 0.7 (0.2) | 0.7 (0.3) | 0.8 (0.1) | 0.001 |
| Iodine (μg/meal) | 120 | 79.3 (33.1) | 81.7 (35.6) | 72.7 (24.2) | 0.025 |
| Selenium (μg/meal) | 39 | 72.4 (21.8) | 73.0 (21.9) | 71.0 (21.8) | 0.542 |
| LA^5^ (g/meal) | 8.9 | 3.1 (1.6) | 3.0 (1.6) | 3.6 (1.5) | 0.004 |
| ALA^6^ (g/meal) | 2.2 | 0.4 (0.4) | 0.5 (0.5) | 0.4 (0.2) | 0.006 |
| DHA^7^ (mg/meal) | 152 | 67.0 (79.5) | 75.4 (90.8) | 43.6 (16.2) | <0.001 |
| SFA^8^ (g/meal) | 26 | 10.1 (5.0) | 10.3 (9.6) | 9.6 (4.2) | 0.317 |
| Salt (g/meal) | 6.5 | 2.4 (0.8) | 2.4 (0.8) | 2.5 (0.8) | 0.325 |
| Total sugars^9^ (g/meal) | 67.5 | 18.6 (6.8) | 17.79 | 20.72 | 0.004 |

^1^ Recommended daily intake for children aged 4–13 years attending primary school in France (Martin, 2001)

^2^ Maximum recommended value for children aged 4-12 years (ANSES, 2012, 2019a, 2021b) ^3^ Mean comparison of vegetarian and non-vegetarian meals (two-sample Student’s t test, significance: *p*<0.05 for meal indicators, *p*<0.002 for nutrients). ^4^Vitamin A = retinol + beta-carotene/6. ^5^Linoleic acid. ^6^Alpha-linolenic acid. ^7^Docosahexaenoic acid. ^8^Saturated fatty acids. ^9^Total sugars = fructose + glucose + maltose + saccharose

**Table S5**. MAR and nutrient content per meal for the five meal subcategories based on protein dish: beef, veal, lamb (n = 56); pork and poultry (n = 68); fish (n = 55); eggs and/or cheese (n = 40); vegan (n = 30), served in the Dijon school canteens in 2019

|  |  | **Mean (SD)** | | | | |  |
| --- | --- | --- | --- | --- | --- | --- | --- |
|  | **RDI**^1^ **or MRV**^2^ | **Beef, Veal, Lamb**  **(n = 56)** | **Pork, Poultry  (n = 68)** | **Fish**  **(n = 55)** | **Eggs and/or cheese  (n = 40)** | **Vegan**  **(n = 30)** | ***p*** ^3^ |
| MAR (%) |  | 48,3 (5,9)^ab^ | 49.1 (6.8)^a^ | 50.9 (6.4)^a^ | 48.4 (6.2)^ab^ | 45.3 (4.7)^b^ | 0.003 |
| Proteins (g/meal) | 25 | 30.9 (7.0)^ab^ | 34.1 (8.0)^a^ | 31.7 (6.3)^ab^ | 26.9 (6.2)^b^ | 21.2 (4.6)^c^ | <0.001 |
| Fibers (g/meal) | 13 | 8.3 (2.3)^b^ | 8.6 (3.1)^b^ | 8.1 (2.5)^b^ | 9.5 (3.6)^b^ | 12.2 (3.3)^a^ | <0.001 |
| Vitamin B1 (mg/meal) | 0.8 | 0.3 (0.1)^ab^ | 0.4 (0.3)^a^ | 0.3 (0.1)^b^ | 0.3 (0.1)^ab^ | 0.3 (0.1)^ab^ | <0.001 |
| Vitamin B2 (mg/meal) | 1.2 | 0.4 (0.1)^ab^ | 0.4 (0.2)^ab^ | 0.4 (0.1)^bc^ | 0.5 (0.2)^a^ | 0.3 (0.1)^c^ | <0.001 |
| Vitamin B3 (mg/meal) | 9 | 5.4 (1.6)^b^ | 8.1 (2.9)^a^ | 4.4 (2.4)^b^ | 3.8 (2.3)^bc^ | 2.4 (0.9)^c^ | <0.001 |
| Vitamin B6 (mg/meal) | 1 | 0.6 (0.2)^ab^ | 0.7 (0.3)^a^ | 0.5 (0.2)^b^ | 0.5 (0.1)^b^ | 0.5 (0.2)^ab^ | <0.001 |
| Vitamin B9 (μg/meal) | 201 | 97.5 (28.7)^c^ | 104.6 (54.9)^bc^ | 106.8 (33.8)^bc^ | 160.3 (88.5)^a^ | 147.1 (73.6)^ab^ | <0.001 |
| Vitamin B12 (μg/meal) | 1.4 | 1.9 (0.9)^ab^ | 0.8 (0.3)^c^ | 2.8 (2.4)^a^ | 0.9 (0.2)^bc^ | 0.5 (0.5)^c^ | <0.001 |
| Vitamin C (mg/meal) | 89 | 25.1 (18.4)^a^ | 22.8 (16.4)^a^ | 22.3 (19.1)^a^ | 22.0 (13.8)^a^ | 18.1 (14.5)^a^ | 0.486 |
| Vitamin D (μg/meal) | 5 | 0.8 (0.6)^b^ | 1.2 (0.7)^b^ | 2.5 (2.0)^a^ | 1.4 (0.7)^b^ | 0.9 (0.6)^b^ | <0.001 |
| Vitamin E (mg/meal) | 9.1 | 3.7 (1.7)^a^ | 3.9 (2.0)^a^ | 4.4 (1.8)^a^ | 5.0 (2.0)^a^ | 4.5 (1.9)^a^ | 0.016 |
| Vitamin A^4^ (μg/meal) | 501 | 454,4 (451,7)^a^ | 469.5 (489.0)^a^ | 440.9 (434.7)^a^ | 528.8 (616.5)^a^ | 326.9 (298.4)^a^ | 0.516 |
| Calcium (mg/meal) | 924 | 221.1 (82.3)^b^ | 227.0 (74.7)^b^ | 254.7 (81.0)^ab^ | 301.1 (77.3)^a^ | 225.3 (71.1)^b^ | <0.001 |
| Potassium (mg/meal) | 2892 | 952.2 (266.4)^ab^ | 1009.4 (257.6)^a^ | 959.0 (262.8)^ab^ | 782.2 (163.9)^b^ | 924.9 (172.8)^ab^ | <0.001 |
| Iron (mg/meal) | 8.2 | 3.8 (1.7)^a^ | 2.9 (0.9)^b^ | 2.6 (0.9)^b^ | 3.4 (0.9)^ab^ | 3.5 (1.1)^ab^ | <0.001 |
| Magnesium (mg/meal) | 203 | 78.6 (15.0)^b^ | 91.5 (24.9)^ab^ | 96.6 (23.4)^ab^ | 90.1 (26.7)^ab^ | 105.1 (29.1)^a^ | <0.001 |
| Zinc (mg/meal) | 9.2 | 5.5 (2.1)^a^ | 3.3 (1.0)^b^ | 2.4 (0.6)^c^ | 2.9 (0.5)^bc^ | 2.5 (0.5)^bc^ | <0.001 |
| Copper (mg/meal) | 1.2 | 0.6 (0.1)^b^ | 0.7 (0.1)^ab^ | 0.7 (0.4)^ab^ | 0.7 (0.2)^ab^ | 0.8 (0.1)^a^ | 0.006 |
| Iodine (μg/meal) | 120 | 60.1 (19.0)^b^ | 69.8 (20.7)^b^ | 117.6 (37.1)^a^ | 70.7 (26.5)^b^ | 77.6 (19.6)^b^ | <0.001 |
| Selenium (μg/meal) | 39 | 60.3 (16.7)^c^ | 71.5 (20.2)^bc^ | 88.7 (18.9)^a^ | 59.6 (17.4)^c^ | 84.5 (19.1)^ab^ | <0.001 |
| LA^5^ (g/meal)^4^ | 8.9 | 2.8 (1.8)^ab^ | 3.4 (1.5)^ab^ | 2.5 (1.5)^b^ | 3.3 (1.4)^ab^ | 3.8 (1.6)^a^ | 0.001 |
| ALA^6^ (g/meal)^5^ | 2.2 | 0.4 (0.4)^a^ | 0.5 (0.5)^a^ | 0.5 (0.5)^a^ | 0.3 (0.2)^a^ | 0.4 (0.1)^a^ | 0.042 |
| DHA^7^ (mg/meal)^6^ | 152 | 42.6 (88.9)^b^ | 42.8 (16.1)^b^ | 150.3 (105.9)^a^ | 47.3 (19.7)^b^ | 40.7 (10.3)^b^ | <0.001 |
| SFA^8^ (g/meal)^7^ | 26 | 10.5 (5.4)^ab^ | 10.2 (5.4)^ab^ | 10.2 (5.2)^ab^ | 11.9 (3.7)^a^ | 6.8 (3.1)^b^ | 0,001 |
| Salt (g/meal) | 6.5 | 2.1 (0.7)^a^ | 2.5 (1.0)^a^ | 2.5 (0.6)^a^ | 2.7 (0.8)^a^ | 2.2 (0.7)^a^ | 0.004 |
| Total sugars^9^ (g/meal) | 67.5 | 17.7 (6.1)^a^ | 19.4 (6.8)^a^ | 16.2 (6.2)^a^ | 20.3 (8.3)^a^ | 20.2 (5.6)^a^ | 0.009 |

^1^ Recommended daily intake for children aged 4–13 years attending primary school in France (Martin, 2001). ^2^ Maximum recommended value for children aged 4-12 years (ANSES, 2012, 2019a, 2021b). ^3^ Type III fixed effects tests of the subcategory effect in ANOVA models with MAR and nutrient content as dependent variables. The same letters indicate no significant difference between subcategories (post hoc pairwise comparisons, significance: *p*<0.05 for meal indicators, *p*<0.002 for nutrients). ^4^Vitamin A = retinol + beta-carotene/6.^5^Linoleic acid. ^6^Alpha-linolenic acid. ^7^Docosahexaenoic acid. ^8^Saturated fatty acids. ^9^Total sugars = fructose + glucose + maltose + saccharose
